# Perinatal outcomes of babies delivered by second-stage Caesarean section versus vacuum extraction in a resource-poor setting, Nigeria – a retrospective analysis

**DOI:** 10.1186/s12884-020-02995-9

**Published:** 2020-05-14

**Authors:** Paul Eze, Lucky Osaheni Lawani, Raphael Ugochukwu Chikezie, Chukwuemeka Ikechi Ukaegbe, Chukwuemeka Anthony Iyoke

**Affiliations:** 1 Medecins Sans Frontieres OCBA, Barcelona, Spain; 2 Centre for Global Health Research, Usher Institute, University of Edinburgh, Edinburgh, UK; 3 Department of Obstetrics & Gynecology, Federal Teaching Hospital, Abakaliki, Nigeria; 4 Department of Obstetrics & Gynecology, University of Nigeria Teaching Hospital, Enugu, Nigeria

**Keywords:** Assisted vaginal delivery, Vacuum extraction, Neonatal assessment, Caesarean section, Decision to delivery interval, Second stage of labour

## Abstract

**Background:**

To evaluate the perinatal status of neonates delivered by assisted vaginal delivery (AVD) versus second-stage caesarean birth (CS).

**Methods:**

A 5-year retrospective study was conducted in a tertiary hospital. Data was analyzed with IBM SPSS® version 25.0 statistical software using descriptive/inferential statistics.

**Results:**

A total of 559 births met the inclusion criteria; AVD (211; 37.7%) and second-stage CS (348; 62.3%). Over 80% of the women were aged 20–34 years: 185 (87.7%) for the AVD group, and 301 (86.5%) for the second-stage CS group. More than half of the women were parous: 106 (50.2%) for the AVD group, and 184 (52.9%) for the second-stage CS group. The commonest indication for intervention in both groups is delayed second stage: 178 (84.4%) in the AVD group, and 239 (68.9%) in the second-stage CS group. There was a statistically significant difference in decision to delivery interval (DDI) between both groups: 197 (93.4%) women in the AVD group had DDI of less than 30 min and 21 women (6.0%) in the CS group had a DDI of less than 30 min (*p* <  0.001). During the DDI, there were 3 (1.4%) intra-uterine foetal deaths (IUFD) in the AVD and 19 (5.5%) in the CS group (*p* = 0.023). After adjusting for co-variates, there were statistically significant differences between the AVD and CS groups in the foetal death during DDI (*p* = 0.029) and perinatal deaths (*p* = 0.040); but no statistically significant differences in severe perinatal outcomes (*p* = 0.811), APGAR scores at 5th minutes (*p* = 0.355), and admission into the NICU (*p* = 0.946). After adjusting for co-variates, use of AVD was significantly associated with the level of experience of the care provider, with resident (junior) doctors less likely to opt for AVD than CS (aOR = 0.45, 95% CI: 0.29–0.70).

**Conclusion:**

Second-stage CS when compared with AVD was not associated with improved perinatal outcomes. AVD is a practical option for reducing the rising Caesarean delivery rates without compromising the clinical status of the newborn.

## Background

Assisted vaginal delivery (AVD; vacuum extraction) and caesarean section (CS) are both obstetric procedures associated with enormous benefits and some complications to women and newborn babies. Either intervention can be undertaken during the second stage of labour for maternal and fetal indications, ranging from prolonged second stage, fetal distress, maternal exhaustion or maternal medical conditions [1, 2]. The procedure of choice should be individualized and depend on meeting pre-requisite criteria, decision-delivery-interval (DDI) and medico-legal considerations [1].

While caesarean section rates have increased dramatically worldwide in the last decades [3–6], AVD is significantly under-utilized, particularly in sub-Saharan Africa; accounting for a meager 1% of institutional births [7–10]. Indeed, the decreasing trend in the utilization of AVD in low and middle-income countries has largely been attributed to decreasing experience with skills required for AVD [7, 8]. Unnecessary CSs place an unjustified burden on the scarce financial and human resources that barely meet the health needs of low-income countries. Fortunately, there is clear evidence indicating that high caesarean section rates can be offset by using AVD, especially in resource poor settings where aversion for CS is paramount. Costs of CS are exorbitant and non-operational universal health insurance schemes results in catastrophic out-of-pocket payment for service charges [2, 7, 9, 11, 12].

Previously evaluated neonatal outcome of AVD was compared with baseline risk of complications from spontaneous vaginal delivery [1]. In situations, however, with life threatening maternal or fetal emergencies in second stage of labour, spontaneous vaginal delivery may not be the best comparison. Hence the need to compare the immediate newborn status, delivered by either AVD or CS. A prospective cohort study conducted in Uganda demonstrated that births by AVD had better maternal and equivalent perinatal outcomes compared to births by second-stage CS [10]. Given that there has been no similar study conducted in Nigeria or in the wider west African region, the present study was necessary to bridge this gap and provides non-existent local data. This study will hopefully complement already existing quality evidence and help improve decision making in obstetric units in resource-poor settings.

## Methods

### Study design

This was a five-year retrospective study, in which data were extracted from hospital records of women who had AVD and second-stage CS over a 5-year period (01 January 2012 to 31 December 2016) in the Federal Teaching Hospital of Abakaliki (FETHA).

### Study setting

FETHA is a tertiary hospital in Abakaliki, the administrative capital of Ebonyi state. The hospital serves residents of Ebonyi state and other neighbouring states. The obstetrical unit of FETHA has an annual delivery rate of about 2000 births. All high-risk deliveries including AVD and CS were performed by senior resident doctors or consultant obstetricians and attended by neonatologists. The vacuum device used in FETHA is the Kiwi© vacuum extractor (Clinical Innovations, South Murray, Utah, USA). The unit also has an operating theatre which is accessible 24 h every day. Foetal monitoring occurs using Pinard fetoscopes or handheld dopplers. Women’s case notes are written by the attending doctors. Standard practice was to admit all neonates with APGAR score of < 7 into the neonatal intensive care unit (NICU) for observation and/or treatment.

### Participants

Inclusion criteria were women with singleton pregnancy ≥37 weeks gestation, cephalic presentation, second stage of labour, engaged fetal head at station + 2 and + 3, and delivery assisted by vacuum device (Kiwi device®) or second-stage CS. Exclusion criteria were women with twin gestation, non-cephalic presentation, preterm births and women with uterine rupture before the decision to intervene. Women with fresh meconium-stained liquor with cardiotocographic evidence of fetal distress (deceleration, bradycardia, tachycardia or loss of baseline variability) in the first stage of labour were excluded.

### Data collection

Women who had CSD were identified from the operating theatre book, and then their case notes were cross-checked to identify women who had fully dilated cervix before the decision to perform CS was made. Women who had AVD were identified from the delivery room register. Women who had CS due to failed AVD were included as AVD as this was the intention a priori. Data were extracted from the women’s case notes, and the admission and discharge register by three trained junior doctors using a proforma. Missing data were cross-checked with the corresponding nursing shift reports for the day. Socio-demographic data were extracted from the case notes. Other information extracted were Apgar scores (1st minute and 5th minute), adverse events during the interventions, admission into NICU and duration of admission. Indications for AVD and second-stage CS were classified as defined in Table 1. Decision to delivery intervals (DDI) were estimated from partographs, surgical notes, and nursing shift reports. Low Apgar score was defined as APGAR < 7 [13]. Women who experienced intra-uterine fetal death before the decision for intervention was made were excluded from the analysis of perinatal outcomes. Study outcomes were foetal death within the DDI, severe perinatal outcomes, low Apgar score at 5-min, admission into NICU, and perinatal death. Severe perinatal outcome was defined as the presence of any of the following: perinatal death, severe birth injury, 5-min Apgar score <  4, and/or convulsions. Severe birth injury was defined as presence of any of the following: dislocation of the leg, clavicular fracture, intracerebral haemorrhage and/or subgaleal haemorrhage.

**Table 1:** Definitions of indications for second stage intervention

Indication classified as:	If the indication for the intervention as stated in the patients’ medical record is;
**Delayed / Prolonged second stage (second stage of labour > 2 h)**	Prolonged labour, obstructed labour, malposition, borderline or contracted pelvis or inadequate contractions
**Foetal distress**	Foetal distress, abnormal foetal heart rate, foetal tachycardia > 160/min or foetal bradycardia < 100/min
**Maternal exhaustion / fatigue**	Maternal exhaustion or insufficient maternal effort
**Other indications**	Other indication stated

### Statistical analysis

Data were entered into Microsoft Excel (Microsoft, Redmond, WA, USA), cleaned and transferred to SPSS version 25.0 (IBM, Armonk, NY, USA) for statistical analyses. Bivariate analyses were performed to determine associations between baseline maternal and neonatal characteristics and mode of delivery. When both Chi-Square test and Fischer’s Exact Tests were estimated, only the *p*-value of the Fischer’s Exact test is reported. Multivariate logistic regression analyses were performed to estimate adjusted odds ratios (aORs) with 95% confidence interval (CI) for study outcomes, and to also estimate the effect of the doctors’ obstetric experience on the odds of opting for a mode of delivery. *P* <  0.05 was used to define statistical significance and all tests were two-tailed.

## Results

**Fig. 1:**
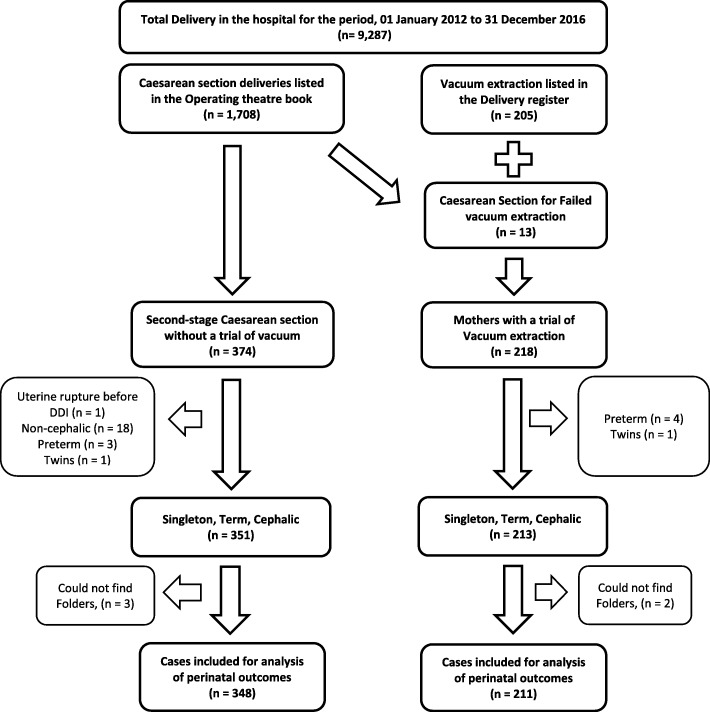
Selection of Cohorts {Cohort 1 – Second stage Caesarean section vs Cohort 2 – Vacuum extraction}

Amongst the 9287 births during the study period, 559 (6.0%) women met the inclusion criteria for the study; 211 (2.3%) with term singleton pregnancies had AVD and 348 (3.7%) women with term singleton pregnancies had second-stage CS. Thirteen women who had CSD due to failed AVD were included in the AVD group (Fig. 1).

Over four-fifth of women in both groups were in the age range 20–34 years and almost half were nulliparous women (Table 2). Other baseline sociodemographic characteristics of the women are shown in Table 2. The commonest indication for both AVD and CS was delayed (or prolonged) second stage of labour: 178 (84.4%) in the AVD group and 239 women (68.7%) in the CS group (*p* <  0.001).

**Table 2:** Baseline characteristics of study cohorts

Characteristics	Assisted Vaginal Delivery (***n*** = 211)	Second-stage Caesarean Delivery (***n*** = 348)	***P***-value
**Mothers’ age**			
− < 20 years	12 (5.7%)	21 (6.0%)	0.916
− 20–34 years	185 (87.7%)	301 (86.5%)	
− ≥ 35 years	14 (6.6%)	26 (7.5%)	
**Marital Status**			
− Single	2 (0.9%)	3 (0.9%)	1.000
− Married	209 (99.1%)	345 (99.1%)	
**Educational qualification**			
− Primary education & below	52 (24.7%)	95 (27.3%)	0.552
− Secondary education & above	159 (75.3%)	253 (72.7%)	
**Religion**			
− Christian	208 (98.6%)	345 (99.1%)	0.677
− Muslim, ATR & others	3 (1.4%)	3 (0.9%)	
**Prenatal care received**			
− ≥ 4 ANC visits	143 (67.8%)	231 (66.4%)	0.942
− 1–3 ANC visits	42 (19.9%)	73 (21.0%)	
− 0 ANC visit	26 (12.3%)	44 (12.6%)	
**Parity**			
− Nulliparous	105 (49.8%)	164 (47.1%)	0.600
− Parous	106 (50.2%)	184 (52.9%)	
**Gestational age at birth**			
− 37^0^–39^6^ weeks	134 (63.5%)	207 (59.5%)	0.372
− ≥ 40 weeks	77 (36.5%)	141 (40.5%)	
**Sex of newborn**			
− Female	107 (50.7%)	169 (48.6%)	0.663
− Male	104 (49.3%)	179 (51.4%)	
**Birth weight**			
− < 2500 g	13 (6.3%)	14 (4.3%)	0.561
− 2500 to 4000 g	178 (85.5%)	285 (86.6%)	
− ≥ 4000 g	17 (8.2%)	30 (9.1%)	
**Indications for intervention**^**a**^			
− Delayed second stage	178 (84.4%)	239 (68.7%)	< 0.001
− Maternal exhaustion / fatigue	49 (23.2%)	63 (18.1%)	0.157
− Foetal distress	6 (2.8%)	71 (20.4%)	< 0.001
− Other indications	0 (0.0%)	8 (2.3%)	0.027
**Imminent Delivery complications**			
− Impending Uterine rupture	3 (1.4%)	9 (2.6%)	0.393
− Placenta abruption	1 (0.5%)	1 (0.3%)	1.000
− Cord prolapse	1 (0.5%)	7 (2.0%)	0.166

**Abbreviations**: *ATR* African traditional religion, *ANC* Antenatal Care consultations

^**a**^ More than one indication could apply

Perinatal outcomes between both groups are shown in Table 3. Compared to 197 (93.4%) women in the AVD group, only 21 women (6.0%) in the CS group had a DDI of less than 30 min (OR 0.01; 95% CI 0.00–0.01). There were 3 (1.4%) intra-uterine foetal deaths (IUFD) during the DDI in the AVD and 19 (5.5%) in the CS group (OR 0.25; 95% CI 0.07–0.85). Perinatal deaths were 7 (3.3%) in the AVD and 27 (7.8%) in the CS (OR 0.41; 95% CI 0.17–0.95) while severe perinatal outcomes were 22 (10.4%) in the AVD group and 42 (12.1%) in the CS group (OR 0.85; 95% CI 0.49–1.47).

**Table 3:** Delivery Outcomes & Perinatal outcomes of Surviving newborns

	Assisted Vaginal Delivery (n = 211)	Second-stage Caesarean Delivery (n = 348)	Crude OR (95% CI)
**Decision to Delivery Interval**			
− < 30 min	197 (93.4%)	21 (6.0%)	0.01 (0.00–0.01)
− ≥ 30 min	14 (6.6%)	327 (94.0%)	
**Outcome of delivery**			
− IUFD during DDI	3 (1.4%)	19 (5.5%)	0.25 (0.07–0.85)
− Perinatal death	7 (3.3%)	27 (7.8%)	0.41 (0.17–0.95)
− Severe Perinatal outcome ^c^	22 (10.4%)	42 (12.1%)	0.85 (0.49–1.47)
**Timing of death**			
− During DDI	3 (1.4%)	19 (5.5%)	0.25 (0.07–0.85)
− Early Neonatal period ^d^	4 (1.9%)	8 (2.3%)	0.82 (0.24–2.76)
**Apgar scores at 5 min**^b^			
− ≥ 7	184 (88.5%)	294 (89.4%)	1.10 (0.63–1.90)
− 4–6 ^a^	17 (8.2%)	29 (8.8%)	
− < 4 ^a^	7 (3.3%)	6 (1.8%)	
**Admission into the NICU**^b^			
− Total admissions	30 (14.4%)	46 (14.0%)	1.04 (0.63–1.70)
− Length of Stay, > 7 days	5 (2.4%)	7 (2.1%)	1.13 (0.36–3.62)
**Adverse events**^b **e**^			
− Birth asphyxia	13 (6.3%)	16 (4.9%)	1.30 (0.61–2.77)
− Convulsions	5 (2.4%)	8 (2.4%)	0.99 (0.32–3.06)
− Jaundice	5 (2.4%)	6 (1.8%)	1.33 (0.40–4.40)
− Sepsis	6 (2.9%)	9 (2.7%)	1.06 (0.37–3.01)
− Breathing difficulties	3 (1.4%)	4 (1.2%)	1.19 (0.26–5.37)
− Feeding difficulties	1 (0.5%)	2 (0.6%)	0.79 (0.07–8.77)
− Severe Birth injury ^f^	3 (1.4%)	1 (0.3%)	4.80 (0.50–46.46)

**Abbreviations**: *OR* Odds Ratio, *CI* Confidence interval, *IUFD* Intrauterine Foetal Death, *DDI* Decision to delivery interval, *NICU* Neonatal Intensive Care Unit

^a^ These two were used as one category for statistics

^b^ Only surviving newborns included for this analysis (Second-stage Caesarean delivery, *n* = 329 & Assisted Vacuum delivery, *n* = 208)

^c^ Severe Perinatal Outcome defined as presence of the following: perinatal death, severe birth injury, 5-min Apgar score < 4, or convulsions

^d^ in the first week after delivery

^e^ More than one adverse event could apply

^f^ Severe Birth injury includes any of the following: dislocation of the leg, clavicular fracture, intracerebral haemorrhage or subgaleal haemorrhage

Table 4 shows the results of multivariate logistics regression analysis for the main study outcomes. After adjusting for parity, booking status, gestational age, indication for the intervention, presence of imminent delivery complications and birthweight, identified from univariate analysis as significant co-variates, statistically significant differences between the AVD and CS groups were detected in foetal deaths during DDI (aOR = 0.22; 95% CI 0.06–0.89) and perinatal deaths (aOR 0.41; 95% CI 0.17–0.96). After adjusting for significant co-variates in a multivariate logistics regression model, the use of AVD was significantly associated with the level of experience of the care provider, with resident doctors less likely to opt for AVD (aOR 0.45; 95% CI 0.29–0.70).

**Table 4:** Multivariate logistics regression analysis of perinatal outcomes

Perinatal outcomes; Assisted vaginal delivery versus second-stage Caesarean delivery	Second-stage Caesarean delivery Adjusted OR (95% CI)	Assisted Vaginal delivery Adjusted OR (95% CI)
**Fetal Death within DDI**		
− Adjusted OR ^a^	1.00 (Reference)	0.22 (0.06–0.89)
**Perinatal death**		
− Adjusted OR ^a^	1.00 (Reference)	0.41 (0.17–0.96)
**Severe Perinatal Outcomes**		
− Adjusted OR ^a^	1.00 (Reference)	1.07 (0.60–1.92)
**Low APGAR score at 5 min**		
− Adjusted OR ^a^	1.00 (Reference)	0.78 (0.46–1.33)
**Admission into NICU**		
− Adjusted OR ^a^	1.00 (Reference)	0.98 (0.58–1.66)
**Qualification/Experience of care provider**^**b**^		
− Adjusted OR ^a^	1.00 (Reference)	0.45 (0.29–0.70)

**Abbreviations**: *OR*, Odds ratio, *CI* Confidence interval, *DDI* Decision to delivery interval

^a^ ORs were adjusted for parity of the women, number of ANC visits received during the pregnancy, gestational age of the pregnancy, indication for the intervention, presence of imminent delivery complication and birthweight of newborn

^b^ Odds of resident (junior) doctors opting for AVD rather than CS

## Discussion

The present study showed that for women who needed assistance in the second stage of labour, there was no significant difference in immediate neonatal outcomes and admission into the NICU for both AVD and CS. This re-affirmed the findings from previous studies which showed no significant difference in immediate neonatal outcomes for AVD and CS [5, 8, 9]. However, it was at variance with a similar study in Israel, where Shmueli et al. reported that CS yielded poorer neonatal outcome than AVD [14].

Incidence of 2.3% AVD in this study is similar to 2.0% in Maiduguri, Nigeria, but higher than 1.5% reported in Enugu, Nigeria and 0.54% reported in Bauchi, Nigeria [10, 15, 16]. Elsewhere in Africa, our incidence of AVD is slightly lower than the 3.1% reported in Kumasi Ghana and 2.8% reported in Kampala, Uganda; but higher than the overall average of about 2.0% reported in rural Tanzania [2, 8, 11, 17]. On the other hand, our incidence of second-stage CS (3.7%) is higher than the 3.3% reported in Kampala, Uganda [10].

The need for interventions to resolve feto-maternal complications in the second stage of labour is necessary. The current study showed that prolonged second stage of labour was the most common indication for 84.4% AVDs and 68.7% CSs. Other researchers have also reported similar indication for AVD and CS [2, 10, 18, 19]. However, other studies have also reported poor maternal effort/exhaustion and fetal distress as major indications for second stage interventions. Opoku et al. reported an incidence of 10.9% for maternal exhaustion and 15.4% for fetal distress in AVD [1, 2].

Several studies have reported low incidence of AVD in many countries, including low-income countries of sub-Saharan Africa [1, 3, 9, 10, 12]. However, the recent increase in Caesarean section rates in many countries has not resulted in any significant improvement in neonatal outcomes [20]. In addition, several high-quality studies demonstrate that the use of AVD in well-selected patients remains a safe and effective method of delivering healthy neonates without compromising the overall birthing experience and outcome [8, 11, 21]. AVD is therefore a safe alternative worldwide, but more so in low resource settings, where there is high aversion to CS due to several socio-cultural reasons [1, 10, 15, 22]. It is therefore disturbing that even for similar indications and similar set of women, resident doctors were less likely to opt for AVD than CS. This is mostly due to decreasing residents doctors’ experience in AVD [1, 10]. If this low usage of AVD persists, it may further aggravate the increasing rates of CS that are already very high in this setting [22]. Hence, given that mothers in this setting have very high aversion to CS and mothers who had AVD report better overall quality of life than mothers who had CS [15, 21], it is imperative that AVD should be encouraged where conditions allow.

Although this study significantly adds to the body of evidence comprehensively contrasting perinatal outcomes for newborns born via AVD with newborns born by second-stage CS, it is not without some limitations. This study did not present similar analysis on maternal outcomes. Also, this study was also performed in one hospital, which could limit it applicability to other settings. There is, therefore, a need for a large multi-centre study with longer follow-up period, incorporating other methods of assessing immediate neonatal outcomes such as arterial blood gas, and qualitative studies to understand the factors driving resident (junior) doctors’ decision-making in second stage events and how these can be tackled to improve usage of AVD in these settings.

## Conclusions

AVD compared with second-stage CS was not associated with worse perinatal morbidity and mortality. Junior doctors are short in confidence in the use of a vacuum device for AVD. With appropriate trainings, AVD could be a practical option in reducing the rising caesarean delivery rates without compromising the clinical status of the newborns.

## Data Availability

The data that support the findings of this study are available from Federal Teaching Hospital Abakaliki, but restrictions apply to the availability of these data, which were used under license for the current study, and so are not publicly available. Data are however available from the authors upon reasonable request and with permission of Federal Teaching Hospital Abakaliki.

## Abbreviations

AVD: Assisted vaginal delivery
CS: Caesarean section
FETHA: Federal Teaching Hospital Abakaliki
NICU: Newborn Intensive Care Unit

## References

[1] Lawani LO, Anozie OB, Ezeonu PO, Iyoke CA (2014). Comparison of outcomes between operative vaginal deliveries and spontaneous vaginal deliveries in Southeast Nigeria. Int J Gynecol Obstet.

[2] Opoku B (2006). A review of vacuum deliveries at komfo anokye teaching hospital, Kumasi. Ghana Med J.

[3] Harrison MS, Saleem S, Ali S, Pasha O, Chomba E, Carlo WA (2019). A prospective, population-based study of trends in operative vaginal delivery compared to cesarean delivery rates in low- and middle-income countries, 2010-2016. Am J Perinatol.

[4] Betrán AP, Ye J, Moller A-B, Zhang J, Gülmezoglu AM, Torloni MR (2016). The increasing trend in caesarean section rates: global, regional and National Estimates: 1990-2014. PLoS One.

[5] Halscott TL, Reddy UM, Landy HJ, Ramsey PS, Iqbal SN, Huang C-C (2015). Maternal and neonatal outcomes by attempted mode of operative delivery from a Low Station in the second stage of labor. Obstet Gynecol.

[6] Erika F, Werner DAS (2012). Mode of delivery and neonatal outcomes in preterm, Small-for- Gestational-Age Newborns. Obs Gynecol.

[7] Nolens B, Capelle M, van Roosmalen J, Mola G, Byamugisha J, Lule J (2019). Use of assisted vaginal birth to reduce unnecessary caesarean sections and improve maternal and perinatal outcomes. Lancet Glob Health.

[8] Nolens B, Namiiro F, Lule J, van den Akker T, van Roosmalen J, Byamugisha J (2018). Prospective cohort study comparing outcomes between vacuum extraction and second-stage cesarean delivery at a Ugandan tertiary referral hospital. Int J Gynecol Obstet.

[9] Bailey P, van Roosmalen J, Mola G, Evans C, de Bernis L, Dao B (2017). Assisted vaginal delivery in low and middle income countries: an overview. BJOG.

[10] Aliyu LD, Kadas AS, Hauwa MA (2011). Instrumental vaginal delivery in Bauchi, Northeast Nigeria. J West African Coll Surg.

[11] Dominico S, Bailey PE, Mwakatundu N, Kasanga M, van Roosmalen J (2018). Reintroducing vacuum extraction in primary health care facilities: a case study from Tanzania. BMC Pregnancy Childbirth.

[12] Ali UA, Norwitz ER (2009). Vacuum-assisted vaginal delivery. Rev Obstet Gynaecol.

[13] Watterberg KL, Aucott S, Benitz WE, Cummings JJ, Eichenwald EC, Goldsmith J (2015). The APGAR score. Pediatrics.

[14] Shmueli A, Salman L, Ashwal E, Hiersch L, Gabbay-Benziv R, Yogev Y (2017). Perinatal outcomes of vacuum assisted versus cesarean deliveries for prolonged second stage of delivery at term*. J Matern Neonatal Med.

[15] Okeke T, Ekwuazi K (2013). Is there still a place for vacuum extraction (ventouse) in modern obstetric practice in Nigeria. Ann Med Health Sci Res.

[16] Mairiga AG, Kyari O, Audu BM, Mairiga (2005). 2005.pdf. Trop J Obstet Gynaecol.

[17] Nolens B, Lule J, Namiiro F, van Roosmalen J, Byamugisha J (2016). Audit of a program to increase the use of vacuum extraction in Mulago hospital, Uganda. BMC Pregnancy Childbirth.

[18] Unterscheider J, McMenamin M, Cullinane F (2011). Rising rates of caesarean deliveries at full cervical dilatation: a concerning trend. Eur J Obstet Gynecol Reprod Biol.

[19] Loudon JAZ, Groom KM, Hinkson L, Harrington D, Paterson-Brown S (2010). Changing trends in operative delivery performed at full dilatation over a 10-year period. J Obstet Gynaecol (Lahore).

[20] Li F, Wu T, Lei X, Zhang H, Mao M, Zhang J (2013). The Apgar score and infant mortality. PLoS One.

[21] Nolens B, van den Akker T, Lule J, Twinomuhangi S, van Roosmalen J, Byamugisha J (2018). Birthing experience and quality of life after vacuum delivery and second-stage caesarean section: a prospective cohort study in Uganda. Trop Med Int Heal.

[22] Sunday-Adeoye I, Kalu CA (2011). Pregnant Nigerian women’s view of cesarean section. Niger J Clin Pract.

